# Are Junior Residents Accurate at Predicting Fetal Weight? An Analysis of Junior Residents' Performance of Estimated Fetal Weight Using Ultrasound and Leopold's Maneuver

**DOI:** 10.1089/whr.2023.0118

**Published:** 2024-02-27

**Authors:** Kimberly Huynh, Alicia Lunardhi, Karren Lewis, Trevor Pickering, Hindi E. Stohl

**Affiliations:** ^1^ Harbor-UCLA Medical Center, Torrance, California, USA.; ^2^ University of Southern California, Los Angeles, California, USA.

**Keywords:** ultrasound, postgraduate education, academic medicine, residency training, obstetrics

## Abstract

**Background:**

Performing accurate estimated fetal weights (EFWs) is a critical skill developed in obstetrics residency training. Resident physicians are often the first to perform EFWs on obstetric patients when they enter care. Evaluating residents' accuracy in performing EFWs is crucial for assessing their achievement in residency training milestones and providing patient care.

**Methods:**

As part of an educational initiative program between 2014 and 2020, postgraduate year 1 (PGY1) and postgraduate year 2 (PGY2) residents performed EFW measurements on 10 term (>37w0d) patients using ultrasound and Leopold's maneuver and 10 preterm (>24w0d and <37w0d) patients using ultrasound. Clinical characteristics, mode of delivery, and actual birthweights (BWs) were recorded for each patient. The accuracy of these estimates was evaluated using mixed-effect regression models.

**Results:**

Thirty-three residents, 1127 deliveries, and 1790 EFW measurements were evaluated. Overall, the percentage of residents with estimations within 10% of actual BW went up in PGY2 for Leopold's and ultrasound term births, but not for preterm ultrasound births. Maternal body mass index and actual BW were associated with absolute percentage estimation error. After adjusting for these variables, there was a statistically significant decrease in error between PGY1 and PGY2 for Leopold's method in term births; ultrasound (term and preterm) showed more modest reductions in error during PGY2.

**Discussion:**

Resident physicians have accurate estimates of EFWs early in their training, beginning in their first year of residency by both Leopold's maneuver and ultrasound. Furthermore, PGY2 residents performed better than PGY1 residents for Leopold's method.

## Introduction

Estimated fetal weight (EFW) is an integral part of obstetric care and is considered a surrogate for gestational age when earlier dating methods are not available. This is often the case for clinicians working in low-resource settings where patients have limited access to care. Often, these patients enter prenatal care in the second or third trimester of their pregnancy and are dated by their ultrasound at their first visit. At academic institutions, obstetrics and gynecology (Ob/Gyn) resident physicians are often the first clinicians these patients interact with and are those performing their dating ultrasounds.

Although EFW is a crucial part of clinical decision making, the existing data are mixed about the differences in the best modality to determine EFWs compared with birthweight (BW). The main methods in modern practice to measure EFWs are fundal height measurements, Leopold's maneuver, and ultrasound.^1,2^ The degree of accuracy is also dependent on the actual fetal weight; there is a consistent tendency to overestimate or underestimate EFWs at the extremes of fetal weight.^3^ There is also mixed evidence on maternal body mass index (BMI) and accuracy of Leopold's maneuver.^2,4–6^

Despite these considerations to calculating an accurate EFW, EFW remains critical to medical decision making in obstetrics. Both low BW and excessive BW are associated with increased risk of newborn and maternal complications during the antepartum period and labor. Thus, EFW dictates prenatal and antepartum counseling, labor management, and delivery modality. Mitigating potential complications during delivery and antepartum management also requires accurate estimation of fetal weight.

As Ob/Gyn residency programs continue to train providers in the field, it is imperative that residents are well trained to assess EFW accurately with dependability. Residents are also often the first provider to patients at these training centers, adding to the importance of EFW in residency curriculum.

Few studies have been done to solely evaluate resident physicians' accuracy in determining EFWs. Of those studies, most suggest that residents' accuracy in determining EFW is independent of their years of experience.^1,7^ Some articles suggest a learning curve and that accuracy increases as resident physicians continue their training,^8^ while others suggest that technological advancements in teaching have dramatically increased proficiency in the first year of residency.^6,9^ These previous studies used a mixture of midwives, physicians, residents, and medical students to perform ultrasound or Leopold's maneuver at various stages in their training. Furthermore, no study has looked at the differences in evaluation of EFWs by residents of preterm and term fetuses.

Although previous studies examined EFW performance using a sample size comprised with mixed practitioners with varying levels of experience (physicians, midwives, residents, and medical students), our study is the first to solely focus on the accuracy of resident physicians. This study explores the accuracy of clinical and sonographic techniques conducted by postgraduate year 1 (PGY1) and postgraduate year 2 (PGY2) resident physicians in preterm and term pregnancy populations. The primary goals of this study were to compare accuracy between resident cohorts and track performance from year 1 to year 2. A secondary goal of this study is evaluating the effect of maternal BMI on the accuracy of EFWs by residents.

## Materials and Methods

### Study design

This is a retrospective observational cohort study involving 35 PGY1 and PGY2 Harbor-UCLA OBGYN resident physicians utilizing a deidentified data set to evaluate resident accuracy of EFWs. As part of a resident education program developed to improve and assess resident clinical skills, residents were observed for their first 2 years of residency. Each year, a participant identified 10 term (>37 weeks) and 10 preterm (<37 weeks) pregnancies for evaluation. The total evaluated were 434 preterm labor subjects and 693 term laboring subjects who presented to the labor and delivery floor. Inclusion criteria were delivery occurring within 2 weeks of the ultrasound biometry and complaint of labor (loss of fluids or contractions). There was no exclusion or withdrawal criteria. The education program has been ongoing since 2014.

### Methodology

Once selected, the resident measured the EFWs by Leopold's maneuver followed by sonography for term pregnancies and sonography alone for preterm pregnancies. Residents recorded the weight measurement on a data collection sheet. The data collection sheet is available as Supplementary Appendix SA1. Additional information recorded by the residents on the data collection sheet included maternal age, gravity and parity, gestation age in weeks and days, and maternal BMI. A resident filled in the actual BW once the fetus was delivered.

We standardized learning of the Leopold's maneuver through resident education training to avoid interpractitioner bias. To standardize sonographic biometry, a 3.5 MHz transducer probe was used to measure biparietal diameter, head circumference, abdominal circumference, and femur length. These measurements were then used in the Hadlock formula to calculate an EFW.

For the study, resident data collection sheets will be consolidated into a master EFW education program study data collection sheet by the primary investigator, Dr. Stohl, to deidentify any resident and patient information. Resident postgraduate year will remain as the identifier. Data will be analyzed to determine whether there is a significant difference between EFWs performed by PGY1 versus PGY2 resident physicians, Leopold's maneuver versus ultrasound in determining EFWs, and EFW accuracy in preterm versus term pregnancies. All data were compared with actual BW to demonstrate the effectiveness of both clinical and sonographic EFWs.

### Statistical analysis

Thirty-three residents, 1127 (434 preterm, 693 term) deliveries, and 1790 estimations were evaluated (1106 ultrasound EFWs, 684 Leopold's EFWs). The accuracy of the residents' EFWs was evaluated as percentage estimation error, which was the difference between estimated BW and actual BW divided by the actual BW. Magnitude of percentage estimation error was computed by taking the absolute value of the percentage estimation error. Mixed-effect regression models with a random intercept for participant were used for statistical modeling of the effect of PGY, adjusting for BMI and actual BW. *p* < 0.05 was considered significant.

## Results

### Sample size characteristics

PGY1s assessed 192 preterm EFWs and 355 term EFWs (reflecting 351 Leopold's EFWs and 346 ultrasound EFWs). PGY2s assessed 242 preterm EFWs and 338 term EFWs (reflecting 333 Leopold's EFWs and 326 ultrasound EFWs). The mean maternal age of preterm fetuses and term fetuses was 28.8 and 27.8 years, respectively. The mean maternal gravida status for preterm fetuses and term fetuses was 3.1 and 2.5, respectively. The mean maternal parity status for preterm fetuses and term fetuses was 1.3 and 1.2, respectively.

The mean gestational age for preterm fetuses when they presented for their EFW assessment was 32.9 weeks for preterm fetuses and 39.0 weeks for term fetuses. The mean maternal BMI was 31.4 kg/m^2^ for preterm fetuses and 32.1 kg/m^2^ for term fetuses. Forty-three percent of the preterm fetuses were delivered through cesarean delivery compared with 33% of term fetuses; 57% of the preterm fetuses were delivered vaginally compared with 67% of the term fetuses. The mean actual BW of preterm fetuses was 2170 g compared with 3386 g for term fetuses (Table 1).

**Table 1.** Descriptive Mean Statistics of Fetuses Delivered at Preterm Versus Term Gestation

Characteristic	Preterm	Term
Postgraduate year		
1	192 (44%)	355 (51%)
2	242 (56%)	338 (49%)
Maternal age	28.8 (7.2)	27.8 (6.1)
Gravida	3.1 (2.3)	2.5 (1.7)
Parity	1.3 (1.6)	1.2 (3.1)
Gestational age (weeks)	32.9 (3.7)	39.0 (1.3)
Body mass index (kg/m^2^)	31.4 (6.8)	32.1 (6.4)
Route of delivery		
Cesarean	182 (43%)	223 (33%)
Vaginal	243 (57%)	452 (67%)
Actual birthweight (g)	2170 (754)	3386 (480)

*n* (%); mean (SD).

SD, standard deviation.

### Findings

**FIG. 1.**
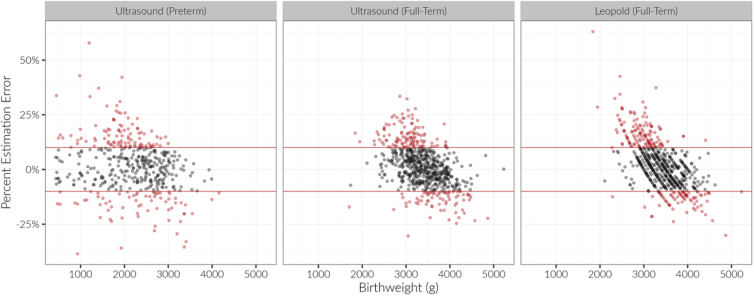
Percentage estimation error vs actual birthweight. Positive values are overestimations while negative values are underestimations. Gray points within the red lines are within 10% of the actual birthweight, while red points are >10% of the actual birthweight.

**FIG. 2.**
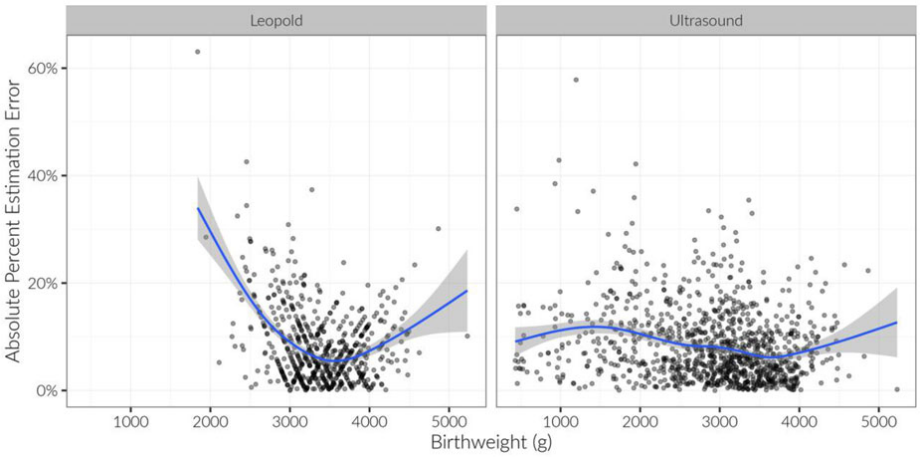
Relationship between actual birthweight and absolute percentage estimation error, by estimation type. A best-fit smoothed line is displayed in each panel.

In total, 61.5% of preterm EFWs (65.6% in PGY1 vs. 58.3% in PGY2), 69.0% of term Leopold's EFWs (68.1% in PGY1 vs. 70.0% in PGY2), and 72.6% of term ultrasound EFWs (69.4% in PGY1 vs. 76.1% in PGY2) were within 10% of actual BW (Fig. 1). Although most residents' EFWs were within 10% of actual BW, there did appear to be a BW at which estimations were more accurate. For ultrasound measurements, the lowest absolute percentage estimation error occurred at 3600–3700 g, and for Leopold's measurements, the lowest absolute percentage estimation error occurred ∼3500 g (Fig. 2). Year of residency, estimation method, actual BW, and term status were not related to accuracy of residents' estimation of BW (*p* > 0.12) (Fig. 1).

For ultrasound estimations, mean percentage error was quite low (+1.3%) across all actual BWs, with 95% of estimations between −19.4% and +22.0% of actual BW. For Leopold's estimations, mean percentage error was also quite low (+1.7%), across all BWs, with 95% of estimations between −18.6% and +22.0% of actual BW. However, Leopold's estimations depended more strongly on actual BW. That is, when actual BW was <3500 g, residents tended to overestimate weight (mean percentage error = +5.9%, confidence interval [95% CI] = −13.8% to +25.5%); when actual BW was >3500 g, residents tended to underestimate BW (mean percentage error = −4.8%, 95% CI = −18.6% to +9.1%).

**FIG. 3.**
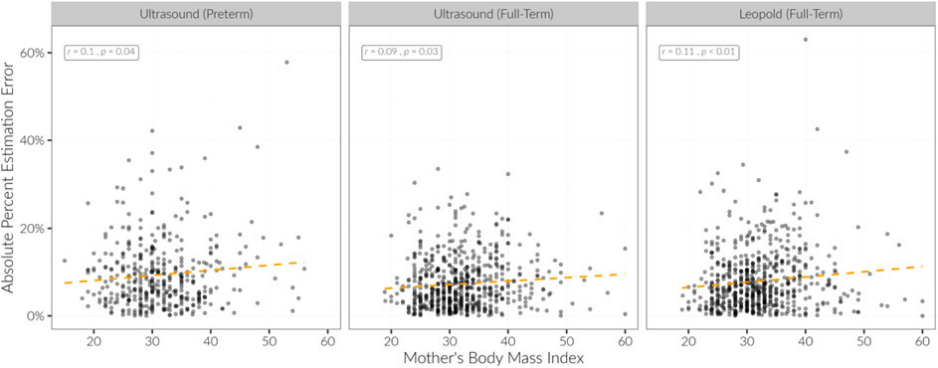
Maternal BMI was associated with decreased accuracy of estimation for all methods. Spearman's correlation coefficients and *p*-values are displayed in each panel, with a best fit linear regression orange dashed line. BMI, body mass index.

Maternal BMI was associated with decreased accuracy of estimation of full-term fetuses for all methods (all *p'*s < 0.05). On average, across all methods, the absolute percentage estimation error increased by 1% for each 10-U increase in maternal BMI (*p* < 0.001) (Fig. 3).

Overall, residents performed more accurate EFWs at term using Leopold's method from PGY1 to PGY2 (Table 2). Adjusting for BMI and BW, residents reduced their percentage error on Leopold's estimations by 1.2% from PGY1 to PGY2 (*p* = 0.01). Although residents reduced their percentage error on ultrasound estimations for term infants (−0.5%) and preterm infants (−0.3%), these effects were not statistically significant (Table 2).

**Table 2.** Comparison of Absolute Percentage Error and Mean Percentage Error Between First- and Second-Year Residents

	Absolute percentage error			Mean percentage error		
Postgraduate year	Term: Leopold's	Term: ultrasound	Preterm: ultrasound	Term: Leopold's	Term: ultrasound	Preterm: ultrasound
1	8.3 (6.4)	7.7 (5.9)	9.6 (8.2)	1.6 (10.4)	0.7 (12.6)	2.1 (9.5)
2	7.7 (7.1)	7.1 (5.8)	9.4 (7.5)	1.8 (10.3)	1.8 (11.9)	0.4 (9.2)

Raw unadjusted value in parentheses. Values were adjusted for maternal BMI and actual birthweight.

BMI, body mass index.

## Discussion

### Interpretation of findings

No previous study has investigated how accurate EFWs are when EFWs are performed exclusively by residents. Educating resident physicians to perform accurate EFWs is a crucial skill taught in training because it drives decisions about mode of delivery and timing of delivery in obstetrics. In addition, this study evaluated the effect of maternal BMI on the accuracy of EFWs by residents.

Based on our results, residents were overall accurate in assessing EFWs early on in their training. This is inconsistent with Noumi et al.'s findings^1^ wherein senior residents' EFWs were more accurate as our study of EFWs performed by PGY1 and PGY2 residents showed satisfactory accuracy for both years.

Our findings that 61.5% of preterm EFWs, 69.0% of term Leopold's EFWs, and 72.6% of term ultrasound EFWs performed by PGY1 and PGY2 residents were within 10% of actual BW are also inconsistent with Predanic et al.'s^8^ findings, suggesting a learning curve exists for ultrasonographic estimates of fetal weight and >70% of estimates were within 10% of BW after 24 months of ultrasonographic experiences. We observed better performance at PGY2 for Leopold's estimations that is consistent with previous studies' findings.^1,6–9^

The lowest estimation error in our study occurred when actual BW was 3600–3700 that is consistent with other studies' findings that predicting BW of 4000 g or more was inaccurate with both methods of estimation.^1^ Lastly, increase in maternal BMI was associated with increased estimation error, which is consistent with previous studies as well.^1,2^

### Strengths and limitations

One strength of our study is our homogeneous sample size. All the EFWs assessed in our study were performed by Ob/Gyn residents in their PGY1 or PGY2 of training as opposed to other studies that assessed EFWs performed by residents, midwives, physicians, and medical students. Also, our data were collected during a real-life clinical situation that was at the time pregnant patients arrived on the labor and delivery floor.

Given that the residents showed overall accurate estimations of EFWs regardless of gestational age and estimation method at an early point in their training, we support residency programs utilizing PGY1 and PGY2 residents performing on patients when they present to labor and delivery. Despite being early on in their training, these residents provided accurate EFWs to inform clinical decision making for antepartum surveillance as well as delivery mode and timing. This further supports how resident physicians continue to be an invaluable and independent member of the patient care team, especially at academic institutions.

A limitation to our study was the small sample size and limiting the analysis of PGY1 and PGY2 residents. Future studies could examine a larger sample size, potentially pooling EFW assessments from multiple cohorts and investigating whether the trend of improvement plateaus at a certain point in training or continues to improve throughout the entire duration of residency. Another limitation of this study is the residents were allowed to choose which patients were included into their list of 10 patients as they presented to the labor and delivery floor that introduces selection bias.

Residents were allowed to pick out their cases from a larger sample of performed estimations and thus risk picking their best (or worst) estimations for evaluation, leading to an under- or overestimated estimation error. If the resident evaluations that were evaluated were randomly drawn, this could have been mitigated. This could falsely skew the residents' performance as being more accurate if they knew the patient prior such as a known history of fetal growth restriction that had been monitored with growth ultrasound on an outpatient basis. Ideally, the residents would have been blinded to the patients they were performing EFWs on, however, this is not realistic in a clinical care setting.

Residents had greater prediction error with higher maternal BMI for term fetuses but not for preterm fetuses, which is consistent with clinical practice where maternal BMI typically peaks near term gestational age. However, this study was conducted in a safety-net training hospital where average maternal BMI is higher than other clinical settings. In addition, many of these patients struggle with access to prenatal care and do not enter care until the third trimester. In this clinical setting, residents are often the first health care providers who pregnant patients encounter and are the first to perform an EFWs.

In this low-resource setting, it is imperative that residents are evaluated in their accuracy and supported educationally to perform accurate EFWs early on in their training to provide the standard of care to patients. An area of interest for future studies would be to compare residents' performance of EFWs in private versus safety-net hospital settings at preterm and term. Another area of interest for future studies would be to test educational initiatives that aimed to improve resident performance of EFWs on higher maternal BMI for term fetuses and analyze the pre- and posteffects of these initiatives.

## Conclusions

In this retrospective cohort study, we found that most residents perform accurate estimations early in their training whether by Leopold's or by ultrasound, regardless of preterm or term status of the fetus. This was demonstrated by the fact that 61.5% of preterm EFWs, 69.0% of term Leopold's EFWs, and 72.6% of term ultrasound EFWs were within 10% of actual BW. Although resident physicians' EFWs were overall accurate, we found that variables not related to residents' training were associated with accuracy, namely, maternal BMI and child's BW.

Lowest absolute percentage estimation error occurred between 3600 and 3700 g. Although residents demonstrated accuracy early on in their training, there was an additional overall trend of improvement in the accuracy of EFWs from PGY1 to PGY2, particularly for Leopold's method. This study provides support for resident physicians to perform EFWs independently early on in their career without compromising patient care.

## Supplementary Material

Supplemental data

## Supplementary Material

Supplementary Appendix SA1

## Abbreviations Used

EFWs: estimated fetal weights
PGY: postgraduate year 1
BW: birthweight

## References

[1] Noumi G, Collado-Khoury F, Bombard A, et al. Clinical and sonographic estimation of fetal weight performed during labor by residents. Am J Obstet Gynecol 2005;192(5):1407–1409; doi: 10.1016/j.ajog.2004.12.043 15902122

[2] Preyer O, Husslein H, Concin N, et al. Fetal weight estimation at term—Ultrasound versus clinical examination with Leopold's manoeuvres: A prospective blinded observational study. BMC Pregnancy Childbirth 2019;19(1):122; doi: 10.1186/s12884-019-2251-5 30971199 PMC6458793

[3] Chauhan SP, Hendrix NW, Magann EF, et al. Limitations of clinical and sonographic estimates of birth weight: Experience with 1034 parturients. Obstet Gynecol 1998;91(1):72–77; doi: 10.1016/s0029-7844(97)00590-5 9464724

[4] Goetzinger KR, Odibo AO, Shanks AL, et al. Clinical accuracy of estimated fetal weight in term pregnancies in a teaching hospital. J Matern Fetal Neonatal Med 2014;27(1):89–93; doi: 10.3109/14767058.2013.806474 23687973 PMC3929500

[5] Gonzalez MG, Reed KL, Center KE, et al. Does maternal body mass index have an effect on the accuracy of ultrasound-derived estimated birth weight?: A retrospective study. J Ultrasound Med 2017;36(5):1009–1014; doi: 10.7863/ultra.16.02073 28258596

[6] Lanowski J-S, Lanowski G, Schippert C, et al. Ultrasound versus clinical examination to estimate fetal weight at term. Geburtshilfe Frauenheilkd 2017;77(3):276–283; doi: 10.1055/s-0043-102406 28392581 PMC5383430

[7] Weiner E, Mizrachi Y, Fainstein N, et al. Comparison between three methods of fetal weight estimation during the active stage of labor performed by residents: A prospective cohort study. Fetal Diagn Ther 2017;42(2):117–123; doi: 10.1159/000450944 27794565

[8] Predanic M, Cho A, Ingrid F, et al. Ultrasonographic estimation of fetal weight: Acquiring accuracy in residency. J Ultrasound Med 2002;21(5):495–500; doi: 10.7863/jum.2002.21.5.495 12008811

[9] Nahum GG. Predicting fetal weight. Are Leopold's maneuvers still worth teaching to medical students and house staff? J Reprod Med 2002;47(4):271–278. 12012878

