# Supplementary figures and images for: Are Junior Residents Accurate at Predicting Fetal Weight? An Analysis of Junior Residents' Performance of Estimated Fetal Weight Using Ultrasound and Leopold's Maneuver
Source: Womens Health Rep (New Rochelle). 2024 Feb 27;5(1):186–92. doi: 10.1089/whr.2023.0118 (PMC10898235; doi:10.1089/whr.2023.0118)

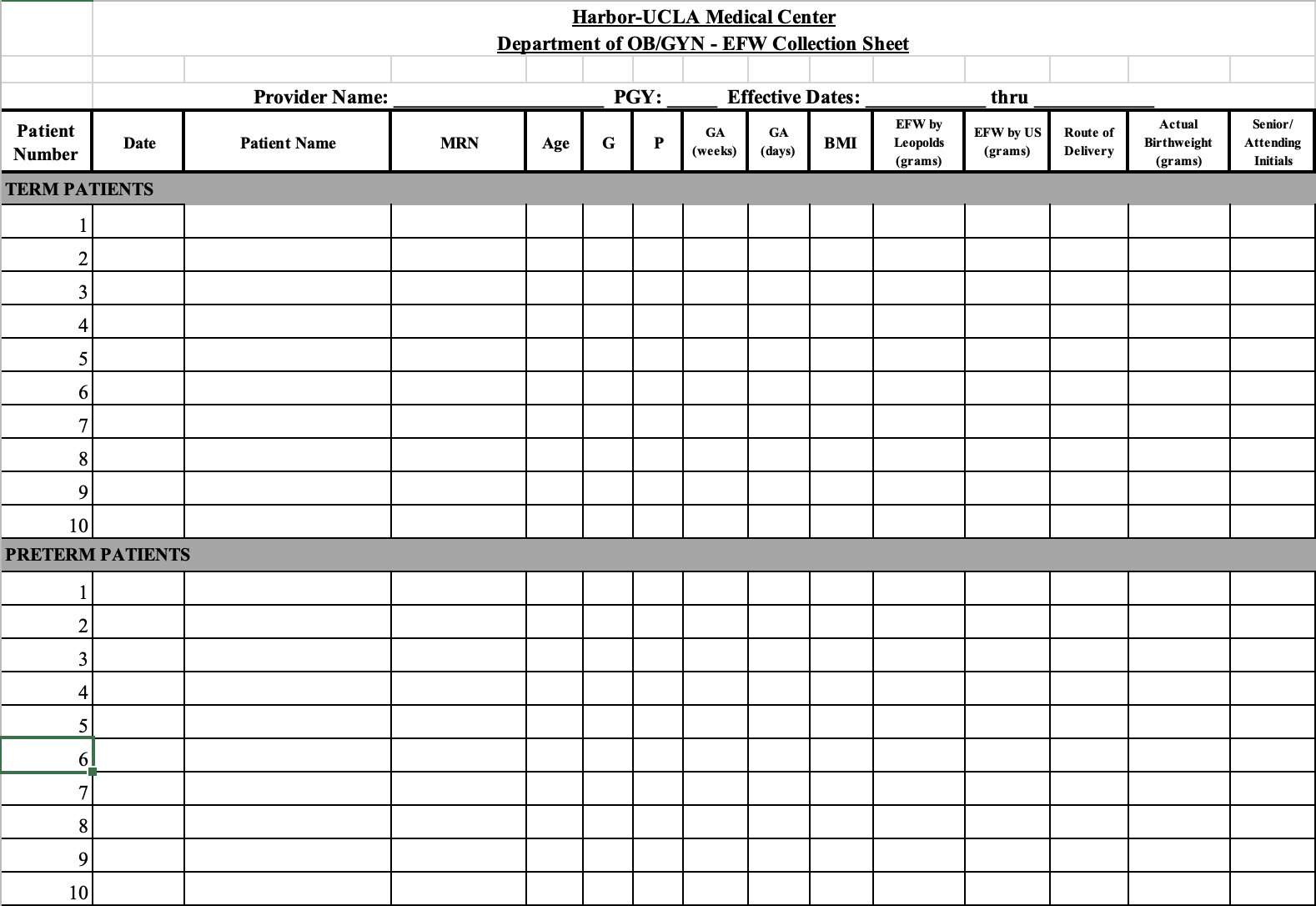

Supplement: Supplemental data [file Suppl_AppendixSA1.docx]
